# Bis(dicyclo­hexyl­phenyl­phosphine)silver(I) nitrate

**DOI:** 10.1107/S1600536810011724

**Published:** 2010-04-10

**Authors:** Andrew R. Burgoyne, Reinout Meijboom, Alfred Muller, Bernard Omondi

**Affiliations:** a Synthesis and Catalysis Research Centre, Department of Chemistry, University of Johannesburg, PO Box 524, Auckland Park, Johannesburg, South Africa 2006

## Abstract

The title compound, [Ag(C_18_H_27_P)_2_]NO_3_, is a mononuclear salt species in which the Ag atom is coordinated by two phosphine ligands, forming a cation, with the nitrate as the counter-anion, weakly inter­acting with the Ag atom, resulting in Ag⋯O distances of 2.602 (6) and 2.679 (6) Å. The cationic silver–phosphine complex has a non-linear geometry in which the P—Ag—P angle is 154.662 (19)°. The Ag—P bond lengths are 2.4303 (6) and 2.4046 (5) Å.

## Related literature

For a review of the chemistry of silver(I) complexes, see: Meijboom *et al.* (2009 ▶). For the coordination chemistry of Ag*X* salts (*X* = F^−^, Cl^−^, Br^−^, I^−^, BF_4_^−^, PF_6_^−^, NO_3_^−^ *etc*) with group 15 donor ligands, with the main focus on tertiary phosphines and in their context as potential anti­tumor agents, see: Berners-Price *et al.* (1998 ▶); Liu *et al.* (2008 ▶). For two- and three-coordinate Ag*X* (*X* = NO_3_^−^) complexes/salts with bulky phosphine ligands, see: Bowmaker *et al.* (1996 ▶); Camalli & Caruso (1988 ▶); Fenske *et al.* (2007 ▶); for *X* = NO_2_, see: Cingolani *et al.* (2002 ▶); for *X* = Cl^−^, Br^−^, I^−^, CN^−^, SCN^−^ and NCO^-^, see: Bowmaker *et al.* (1996 ▶); Bayler *et al.* (1996 ▶); and for two coordinate *X* = ClO_4_^-^, see: Alyea *et al.* (1982 ▶, 2002 ▶); Baiada *et al.* (1990 ▶). For the solution behavior of [*L_n_*Ag*X*] complexes, see: Muetterties & Alegranti (1972 ▶). For atomic radii, see: Pauling (1960 ▶).
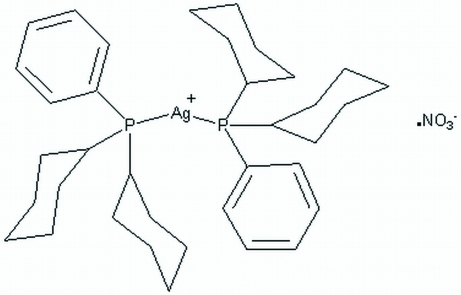


## Experimental

### 

#### Crystal data

[Ag(C_18_H_27_P)_2_]NO_3_
*M* *_r_* = 718.61
Monoclinic, 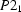

*a* = 10.9207 (4) Å
*b* = 13.6312 (5) Å
*c* = 12.2121 (5) Å
β = 106.896 (1)°
*V* = 1739.45 (11) Å^3^
*Z* = 2
Mo *K*α radiation
μ = 0.71 mm^−1^
*T* = 296 K
0.42 × 0.34 × 0.14 mm

#### Data collection

Bruker APEXII CCD diffractometer
Absorption correction: multi-scan (*SADABS*; Bruker, 2009 ▶) *T*_min_ = 0.756, *T*_max_ = 0.908
19954 measured reflections
6614 independent reflections
6503 reflections with *I* > 2σ(*I*)
*R*_int_ = 0.020

#### Refinement

*R*[*F* ^2^ > 2σ(*F* ^2^)] = 0.021
*wR*(*F* ^2^) = 0.055
*S* = 1.06
6614 reflections
389 parameters
1 restraint
H-atom parameters constrained
Δρ_max_ = 0.75 e Å^−3^
Δρ_min_ = −0.28 e Å^−3^
Absolute structure: Flack (1983 ▶), 2322 Friedel pairs
Flack parameter: 0.041 (15)

### 

Data collection: *APEX2* (Bruker, 2009 ▶); cell refinement: *SAINT* (Bruker, 2009 ▶); data reduction: *SAINT*; program(s) used to solve structure: *SHELXS97* (Sheldrick, 2008 ▶); program(s) used to refine structure: *SHELXL97* (Sheldrick, 2008 ▶); molecular graphics: *ORTEP-3 for Windows* (Farrugia, 1997 ▶), *PLATON* (Spek, 2009 ▶) and *DIAMOND* (Brandenburg & Putz, 2005 ▶); software used to prepare material for publication: *WinGX* (Farrugia, 1999 ▶).

## Supplementary Material

Crystal structure: contains datablocks global, I. DOI: 10.1107/S1600536810011724/bg2336sup1.cif

Structure factors: contains datablocks I. DOI: 10.1107/S1600536810011724/bg2336Isup2.hkl

Additional supplementary materials:  crystallographic information; 3D view; checkCIF report

## Figures and Tables

**Fig. 1.**
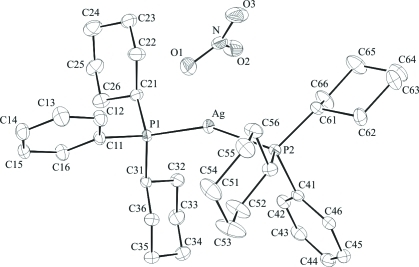
The molecular structure of (I), showing 50% probability displacement ellipsoids. H atoms have been omitted for clarity.

**Fig. 2.**
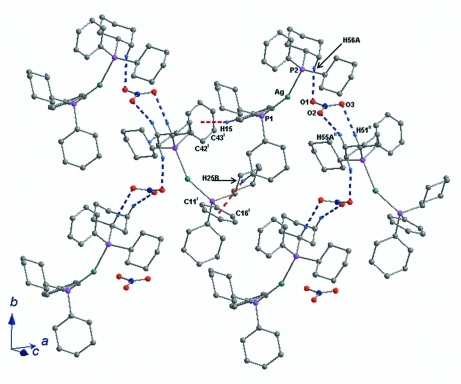
A perspective of (I) where C—H···O and C—H···π intermolecular interactions are shown in dashed lines [Symmetry codes: (i) -x+1, y-1/2, -z+1; (ii) -x+2, -y-1/2, -z+2].

## supplementary crystallographic information

### Comment

Reaction of silver(I) salts with monodentate tertiary phosphines in a 1:2 stoichiometric ratio generally results in the formation of either monomeric [AgX(PR_3_)_2_]/[Ag(PR_3_)_2_]^+^X^-^ or dimeric complexes [{AgX(PR_3_)_2_}_2_] (Meijboom *et al.*, 2009; Bowmaker *et al.*, 1996 and references therein) depending on the donor properties of the phosphine ligand, the bulkiness of the ligand substituents and the donor capabilities of the anion. When π-acid ligands are used in such reactions the complexes formed have been shown to be stable and univalent and these can be two-, three- or four-coordinate depending upon the size and ligation capabilities of the ligands (Baiada *et al.*, 1990). Generally a combination of a weak donor anion and bulky phosphine ligand often leads to the formation of two- or three-coordinate complexes.

The difference between two- and three-coordinate complexes is hinged on the correlation between increasing Ag—P bond distance and decreasing P—Ag—P angle which is determined by the donor properties of the anion (Bowmaker *et al.*, 1996). The longer the interaction between the anion atom/s and the Ag atom, the more linear (closer to 180°) the P—Ag—P angle will be, although the presence of bulky phosphine ligands (such as tricyclohexylphosphine or phenyldicyclohexylphosphine) would also influence the P—Ag—P angle.

The title compound (I) crystallizes in the monoclinic noncentrosymmetric space group P2_1_ and the asymmetric unit contains one Ag(I) cation and one nitrate anionic ligand. The crystal structure of the title compound [Ag{PPh(C_6_H_11_)_2_].NO_3_ (Fig. 1) shows that the complex contains well resolved [Ag{PPh(C_6_H_11_)_2_}_2_]^+^ cation and NO_3_^-^ anion. Examination of the structure with PLATON (Spek, 2009) showed that there were no solvent accessible voids in the crystal lattice.

As shown in Fig. 1, the cation shows a nonlinear coordination sphere in which the P—Ag—P angle is 154.662 (19) °. The NO_3_^-^ anion situated about 2.6 Å away from the Ag center. Similar distortions from linearity have been observed in [Ag{PPh_2_(C_5_H_8_)}_2_]^+^.ClO_4_^-^ (Baiada *et al.*, 1990). The distortion from linearity arises from weak electrostatic interactions of the Ag ion and the nitrate counterion which leads to Ag···O distances of 2.602 and 2.679 Å. In addition the presence of bulky cyclohexyl rings on the phosphine ligands may as well be a contributing factor to the nonlinear behaviour.

The cation Ag—P bond distances are 2.4303 (6) and 2.4046 (5) Å which are well within the Ag—P bond length range for two- or three-coordinate complexes of this type (2.352 -2.521 Å). Comparatively, the Ag—P distances of 2.461 (6) Å (Alyea *et al.*, 1982) and 2.4409 (9) Å (Bayler *et al.*, 1996) have been reported for the bis(trimesitylphosphine) silver(I) cation, an average of 2.416 (2) Å for [Ag{P(C_5_H_9_)Ph_2_}_2_].ClO_4_ (Baiada *et al.*, 1990). Based on the sum of covalent radii of Ag and P atoms, the Ag—P distance is calculated as 2.44 Å (Pauling, 1960).

In the crystal, the Ag^I^ complex interacts with the three nitrate oxygens resulting in C—H···O intermolecular interactions [H51···O3 = 2.46 Å, C51—H51···O3 = 177 °; H55···O2^i ^= 2.53 Å, C55—H55···O2 = 150 ° symmetry code: i: -x, y+1/2, z)] and a C—H···O intramolecular intraction (H56A···O1 = 2.42 Å, C56—H56A···O1 = 150 °). The structure is further stabilized by two C—H···π intermolecular interactions involving the phenyl rings [H25B···Cg1^ii^ = 2.97 Å, C25—H25B···Cg1 = 161° and H15···Cg4^ii^ = 2.85 Å, C15—H15···Cg4 = 151° (Fig. 2). Cg1 and Cg6 are the centroids of the C11/C12/C13/C14/C15/C16 and C41/C42/C43/C44/C45/C46 benzene rings]. Symmetry code for the two interactions, ii: is -x+1, y-1/2, -z+1. The two C—H···π interactions result in dimeric pairs of the the adjacent molecules involved (See Fig 2).

Despite the number of structural reports of [L_n_AgX] complexes, their solution behaviour, initiated by Muetterties & Alegranti (1972), has always shown that the coordinating ligands were labile in all complexes studied. Rapid ligand-exchange reactions have been reported for all ^31^P NMR spectroscopic investigations of ionic Ag^I^ monodentate phosphine complexes, thus making NMR spectroscopy of limited use for these types of complexes.

### Experimental

AgNO_3_ (0.14 g, 0.50 mmol) and P{(C_6_H_11_)_2_Ph} (0.40 g, 1.0 mmol) were dissolved in warm ethanol to give a clear solution which on cooling and solvent evaporation deposited colourless crystals of [Ag{PPh(C_6_H_11_)_2_].^+^NO_3_^-^ in good yield. IR: 699, 745, 1303, 1336, 1387, 2342, 2359, 2849, 2927.

### Refinement

All hydrogen atoms were positioned geometrically, with C–H = 0.98 Å for methine Hydrogens, 0.97 Å for methylene hydrogen and 0.93 Å for aromatic hydrogens, and allowed to ride on their parent atoms with U_iso_(H) = 1.2U_eq_(C).

### Crystal data

**Table:**

[Ag(C_18_H_27_P)_2_]NO_3_	*F*(000) = 756
*M**_r_* = 718.61	*D*_x_ = 1.372 Mg m^−^^3^
Monoclinic, *P*2_1_	Mo *K*α radiation, λ = 0.71073 Å
Hall symbol: P 2yb	Cell parameters from 19966 reflections
*a* = 10.9207 (4) Å	θ = 1.7–27.8°
*b* = 13.6312 (5) Å	µ = 0.71 mm^−^^1^
*c* = 12.2121 (5) Å	*T* = 296 K
β = 106.896 (1)°	Plate, colourless
*V* = 1739.45 (11) Å^3^	0.42 × 0.34 × 0.14 mm
*Z* = 2	

### Data collection

**Table:**

Bruker APEXII CCD diffractometer	6503 reflections with *I* > 2σ(*I*)
Detector resolution: 0 pixels mm^-1^	*R*_int_ = 0.020
φ and ω scans	θ_max_ = 27.8°, θ_min_ = 1.7°
Absorption correction: multi-scan (*SADABS*; Bruker, 2004)	*h* = −14→13
*T*_min_ = 0.756, *T*_max_ = 0.908	*k* = −17→14
19954 measured reflections	*l* = −15→15
6614 independent reflections	

### Refinement

**Table:**

Refinement on *F*^2^	H-atom parameters constrained
Least-squares matrix: full	*w* = 1/[σ^2^(*F*_o_^2^) + (0.0323*P*)^2^ + 0.3975*P*] where *P* = (*F*_o_^2^ + 2*F*_c_^2^)/3
*R*[*F*^2^ > 2σ(*F*^2^)] = 0.021	(Δ/σ)_max_ = 0.001
*wR*(*F*^2^) = 0.055	Δρ_max_ = 0.75 e Å^−^^3^
*S* = 1.06	Δρ_min_ = −0.28 e Å^−^^3^
6614 reflections	Absolute structure: Flack (1983), 2322 Friedel pairs
389 parameters	Flack parameter: 0.041 (15)
1 restraint	

### Special details

**Table:**

Geometry. All esds (except the esd in the dihedral angle between two l.s. planes) are estimated using the full covariance matrix. The cell esds are taken into account individually in the estimation of esds in distances, angles and torsion angles; correlations between esds in cell parameters are only used when they are defined by crystal symmetry. An approximate (isotropic) treatment of cell esds is used for estimating esds involving l.s. planes.

### Fractional atomic coordinates and isotropic or equivalent isotropic displacement parameters (Å^2^)

**Table:**

	*x*	*y*	*z*	*U*_iso_*/*U*_eq_	
C11	0.62500 (19)	0.63257 (16)	0.56623 (17)	0.0192 (4)	
C12	0.7541 (2)	0.65504 (18)	0.57934 (19)	0.0238 (4)	
H12	0.8061	0.6781	0.649	0.029*	
C13	0.8040 (2)	0.6424 (2)	0.4866 (2)	0.0304 (5)	
H13	0.8877	0.6614	0.4936	0.036*	
C14	0.7305 (2)	0.60220 (18)	0.38554 (19)	0.0247 (5)	
H14	0.7665	0.5895	0.3267	0.03*	
C15	0.6018 (2)	0.58046 (17)	0.37122 (18)	0.0216 (4)	
H15	0.55	0.5578	0.3012	0.026*	
C16	0.5517 (2)	0.59328 (18)	0.46349 (19)	0.0230 (4)	
H16	0.4675	0.575	0.4557	0.028*	
C21	0.5685 (2)	0.51594 (16)	0.74331 (19)	0.0209 (4)	
H21	0.5338	0.5188	0.8088	0.025*	
C22	0.7053 (2)	0.47939 (19)	0.7908 (2)	0.0297 (5)	
H22A	0.7475	0.4834	0.7312	0.036*	
H22B	0.7508	0.5221	0.8528	0.036*	
C23	0.7135 (3)	0.3754 (2)	0.8343 (2)	0.0347 (6)	
H23A	0.6873	0.3738	0.9036	0.042*	
H23B	0.8018	0.3537	0.8536	0.042*	
C24	0.6306 (3)	0.30502 (19)	0.7477 (3)	0.0379 (6)	
H24A	0.6652	0.2982	0.6834	0.046*	
H24B	0.6323	0.241	0.7827	0.046*	
C25	0.4941 (3)	0.34062 (19)	0.7054 (2)	0.0344 (6)	
H25A	0.4557	0.3375	0.7676	0.041*	
H25B	0.4461	0.2973	0.6451	0.041*	
C26	0.4845 (2)	0.44473 (18)	0.6600 (2)	0.0283 (5)	
H26A	0.5085	0.4454	0.5895	0.034*	
H26B	0.3963	0.4664	0.6422	0.034*	
C31	0.40036 (18)	0.68109 (17)	0.63811 (18)	0.0175 (4)	
H31	0.3571	0.6355	0.577	0.021*	
C32	0.3332 (2)	0.67641 (18)	0.7323 (2)	0.0236 (4)	
H32A	0.3781	0.7179	0.7959	0.028*	
H32B	0.3354	0.6096	0.7603	0.028*	
C33	0.1933 (2)	0.7106 (2)	0.6862 (2)	0.0346 (6)	
H33A	0.1464	0.6653	0.6278	0.042*	
H33B	0.1539	0.7104	0.7478	0.042*	
C34	0.1855 (3)	0.8127 (2)	0.6359 (2)	0.0354 (6)	
H34A	0.2253	0.859	0.6961	0.042*	
H34B	0.0964	0.8312	0.6046	0.042*	
C35	0.2518 (2)	0.8184 (2)	0.5419 (2)	0.0323 (5)	
H35A	0.2495	0.8855	0.5148	0.039*	
H35B	0.2067	0.7775	0.478	0.039*	
C36	0.3913 (2)	0.78417 (17)	0.5874 (2)	0.0253 (5)	
H36A	0.4303	0.7846	0.5255	0.03*	
H36B	0.4384	0.8295	0.6457	0.03*	
C41	0.61465 (18)	0.98163 (16)	0.93476 (17)	0.0170 (4)	
C42	0.4998 (2)	0.93063 (17)	0.91929 (18)	0.0210 (4)	
H42	0.4969	0.8635	0.9052	0.025*	
C43	0.3901 (2)	0.97931 (19)	0.92474 (19)	0.0257 (5)	
H43	0.3146	0.9445	0.9157	0.031*	
C44	0.3926 (2)	1.07889 (19)	0.94354 (19)	0.0267 (5)	
H44	0.3188	1.1113	0.9466	0.032*	
C45	0.5064 (2)	1.13139 (18)	0.95813 (19)	0.0247 (5)	
H45	0.5079	1.1989	0.9697	0.03*	
C46	0.6173 (2)	1.08229 (17)	0.95532 (18)	0.0199 (4)	
H46	0.6935	1.1168	0.9672	0.024*	
C51	0.84078 (19)	0.99516 (16)	0.85746 (18)	0.0192 (4)	
H51	0.8576	1.0564	0.9014	0.023*	
C52	0.7564 (2)	1.0186 (2)	0.7380 (2)	0.0359 (6)	
H52A	0.6781	1.0494	0.7428	0.043*	
H52B	0.7336	0.9582	0.6948	0.043*	
C53	0.8250 (2)	1.0873 (3)	0.6755 (3)	0.0463 (8)	
H53A	0.7701	1.1001	0.5988	0.056*	
H53B	0.8431	1.1493	0.7159	0.056*	
C54	0.9503 (2)	1.0404 (2)	0.6691 (2)	0.0347 (6)	
H54A	0.932	0.9798	0.6259	0.042*	
H54B	0.9936	1.0844	0.6301	0.042*	
C55	1.0355 (2)	1.0198 (2)	0.7882 (2)	0.0283 (5)	
H55A	1.0578	1.0812	0.8294	0.034*	
H55B	1.114	0.9892	0.7835	0.034*	
C56	0.9694 (2)	0.95221 (19)	0.8541 (2)	0.0252 (5)	
H56A	0.9555	0.8883	0.8175	0.03*	
H56B	1.0244	0.9435	0.9316	0.03*	
C61	0.85318 (19)	0.89667 (16)	1.07819 (17)	0.0176 (4)	
H61	0.9256	0.8543	1.0778	0.021*	
C62	0.9082 (2)	0.98983 (17)	1.1412 (2)	0.0248 (4)	
H62A	0.8391	1.0351	1.1394	0.03*	
H62B	0.9641	1.0208	1.1025	0.03*	
C63	0.9839 (2)	0.9693 (2)	1.2660 (2)	0.0297 (5)	
H63A	1.0594	0.9311	1.2679	0.036*	
H63B	1.012	1.031	1.3048	0.036*	
C64	0.9043 (2)	0.9140 (2)	1.3288 (2)	0.0323 (5)	
H64A	0.957	0.8982	1.4055	0.039*	
H64B	0.8346	0.9555	1.3355	0.039*	
C65	0.8500 (3)	0.8203 (2)	1.2667 (2)	0.0380 (6)	
H65A	0.7951	0.7888	1.3061	0.046*	
H65B	0.9194	0.7756	1.2677	0.046*	
C66	0.7729 (2)	0.8412 (2)	1.1425 (2)	0.0322 (6)	
H66A	0.6981	0.8799	1.1416	0.039*	
H66B	0.7437	0.7797	1.1036	0.039*	
P1	0.56910 (5)	0.64378 (4)	0.69333 (4)	0.01558 (10)	
P2	0.75517 (5)	0.91135 (4)	0.92785 (4)	0.01542 (10)	
Ag	0.702066 (12)	0.754956 (13)	0.830004 (11)	0.01948 (4)	
N	0.9763 (2)	0.69958 (15)	0.93701 (19)	0.0315 (5)	
O1	0.94184 (16)	0.71192 (15)	0.83128 (16)	0.0352 (4)	
O2	0.8923 (2)	0.67588 (16)	0.98484 (17)	0.0425 (5)	
O3	1.08964 (19)	0.71145 (16)	0.9931 (2)	0.0535 (6)	

### Atomic displacement parameters (Å^2^)

**Table:**

	*U*^11^	*U*^22^	*U*^33^	*U*^12^	*U*^13^	*U*^23^
C11	0.0215 (9)	0.0226 (11)	0.0146 (10)	0.0002 (8)	0.0069 (8)	−0.0013 (8)
C12	0.0194 (9)	0.0303 (12)	0.0206 (11)	−0.0020 (9)	0.0041 (8)	−0.0032 (9)
C13	0.0204 (10)	0.0448 (15)	0.0277 (12)	−0.0036 (10)	0.0099 (9)	−0.0033 (11)
C14	0.0276 (10)	0.0296 (12)	0.0199 (11)	−0.0002 (9)	0.0114 (9)	−0.0007 (9)
C15	0.0229 (10)	0.0249 (11)	0.0154 (10)	−0.0001 (9)	0.0029 (8)	−0.0039 (8)
C16	0.0185 (9)	0.0301 (12)	0.0203 (11)	−0.0037 (9)	0.0055 (8)	−0.0029 (9)
C21	0.0238 (10)	0.0192 (10)	0.0203 (11)	0.0045 (8)	0.0073 (8)	0.0018 (8)
C22	0.0261 (11)	0.0269 (12)	0.0325 (13)	0.0049 (9)	0.0026 (10)	0.0035 (11)
C23	0.0449 (14)	0.0338 (15)	0.0279 (13)	0.0170 (11)	0.0142 (11)	0.0062 (10)
C24	0.0504 (16)	0.0240 (14)	0.0440 (16)	0.0075 (11)	0.0210 (13)	0.0112 (11)
C25	0.0402 (13)	0.0242 (13)	0.0445 (15)	−0.0002 (10)	0.0214 (12)	0.0032 (11)
C26	0.0263 (11)	0.0217 (12)	0.0376 (14)	−0.0022 (9)	0.0103 (10)	0.0026 (10)
C31	0.0136 (8)	0.0222 (11)	0.0150 (10)	0.0021 (7)	0.0015 (7)	−0.0026 (8)
C32	0.0208 (9)	0.0289 (12)	0.0228 (11)	0.0036 (8)	0.0092 (9)	0.0020 (9)
C33	0.0237 (11)	0.0471 (15)	0.0351 (15)	0.0079 (11)	0.0117 (11)	−0.0034 (12)
C34	0.0311 (12)	0.0393 (15)	0.0336 (14)	0.0166 (11)	0.0061 (11)	−0.0034 (12)
C35	0.0372 (13)	0.0303 (13)	0.0253 (12)	0.0159 (11)	0.0027 (10)	0.0018 (10)
C36	0.0288 (11)	0.0240 (12)	0.0222 (11)	0.0065 (8)	0.0058 (9)	0.0036 (8)
C41	0.0172 (9)	0.0208 (10)	0.0125 (9)	0.0037 (8)	0.0037 (7)	0.0031 (8)
C42	0.0217 (10)	0.0235 (11)	0.0168 (10)	−0.0012 (8)	0.0041 (8)	0.0017 (8)
C43	0.0181 (9)	0.0376 (14)	0.0213 (11)	0.0009 (9)	0.0057 (8)	0.0037 (10)
C44	0.0246 (10)	0.0363 (14)	0.0192 (11)	0.0111 (10)	0.0065 (9)	0.0027 (10)
C45	0.0313 (11)	0.0251 (11)	0.0173 (10)	0.0080 (9)	0.0062 (9)	−0.0002 (9)
C46	0.0209 (9)	0.0210 (11)	0.0153 (10)	0.0019 (8)	0.0010 (8)	0.0007 (8)
C51	0.0200 (9)	0.0202 (10)	0.0192 (10)	0.0014 (8)	0.0088 (8)	0.0044 (8)
C52	0.0186 (10)	0.0614 (19)	0.0267 (13)	0.0010 (11)	0.0049 (9)	0.0190 (12)
C53	0.0272 (11)	0.075 (2)	0.0376 (16)	0.0090 (14)	0.0113 (11)	0.0342 (15)
C54	0.0247 (11)	0.0609 (18)	0.0195 (12)	−0.0028 (11)	0.0081 (10)	0.0086 (11)
C55	0.0198 (10)	0.0432 (15)	0.0230 (13)	−0.0013 (10)	0.0081 (9)	0.0018 (11)
C56	0.0202 (10)	0.0330 (13)	0.0224 (11)	0.0049 (9)	0.0061 (9)	0.0063 (9)
C61	0.0161 (9)	0.0187 (10)	0.0158 (10)	−0.0012 (7)	0.0014 (7)	0.0014 (8)
C62	0.0333 (11)	0.0213 (11)	0.0179 (11)	−0.0080 (9)	0.0043 (9)	−0.0013 (9)
C63	0.0291 (11)	0.0372 (14)	0.0196 (12)	−0.0083 (10)	0.0018 (10)	−0.0036 (10)
C64	0.0310 (12)	0.0481 (16)	0.0164 (11)	0.0002 (11)	0.0045 (9)	0.0058 (11)
C65	0.0402 (14)	0.0427 (16)	0.0261 (13)	−0.0098 (12)	0.0018 (11)	0.0140 (12)
C66	0.0345 (12)	0.0365 (15)	0.0207 (12)	−0.0152 (11)	0.0005 (10)	0.0089 (10)
P1	0.0160 (2)	0.0174 (3)	0.0130 (2)	0.00103 (18)	0.00354 (18)	−0.00190 (19)
P2	0.0160 (2)	0.0154 (2)	0.0141 (2)	0.00057 (19)	0.00320 (19)	0.00052 (19)
Ag	0.02057 (7)	0.01953 (7)	0.01629 (7)	−0.00042 (7)	0.00212 (5)	−0.00397 (7)
N	0.0290 (10)	0.0190 (11)	0.0357 (12)	0.0087 (8)	−0.0075 (9)	−0.0074 (8)
O1	0.0291 (8)	0.0424 (11)	0.0296 (10)	0.0055 (7)	0.0012 (7)	−0.0091 (8)
O2	0.0540 (12)	0.0315 (10)	0.0356 (11)	0.0093 (9)	0.0030 (9)	0.0070 (8)
O3	0.0350 (10)	0.0314 (11)	0.0684 (15)	0.0080 (8)	−0.0255 (10)	−0.0150 (10)

### Geometric parameters (Å, °)

**Table:**

C11—C16	1.385 (3)	C42—C43	1.389 (3)
C11—C12	1.406 (3)	C42—H42	0.93
C11—P1	1.832 (2)	C43—C44	1.376 (4)
C12—C13	1.402 (3)	C43—H43	0.93
C12—H12	0.93	C44—C45	1.399 (3)
C13—C14	1.376 (3)	C44—H44	0.93
C13—H13	0.93	C45—C46	1.393 (3)
C14—C15	1.397 (3)	C45—H45	0.93
C14—H14	0.93	C46—H46	0.93
C15—C16	1.399 (3)	C51—C52	1.516 (3)
C15—H15	0.93	C51—C56	1.534 (3)
C16—H16	0.93	C51—P2	1.840 (2)
C21—C26	1.509 (3)	C51—H51	0.98
C21—C22	1.520 (3)	C52—C53	1.534 (4)
C21—P1	1.847 (2)	C52—H52A	0.97
C21—H21	0.98	C52—H52B	0.97
C22—C23	1.508 (4)	C53—C54	1.532 (4)
C22—H22A	0.97	C53—H53A	0.97
C22—H22B	0.97	C53—H53B	0.97
C23—C24	1.516 (4)	C54—C55	1.508 (3)
C23—H23A	0.97	C54—H54A	0.97
C23—H23B	0.97	C54—H54B	0.97
C24—C25	1.509 (4)	C55—C56	1.534 (3)
C24—H24A	0.97	C55—H55A	0.97
C24—H24B	0.97	C55—H55B	0.97
C25—C26	1.516 (3)	C56—H56A	0.97
C25—H25A	0.97	C56—H56B	0.97
C25—H25B	0.97	C61—C62	1.516 (3)
C26—H26A	0.97	C61—C66	1.535 (3)
C26—H26B	0.97	C61—P2	1.848 (2)
C31—C36	1.527 (3)	C61—H61	0.98
C31—C32	1.535 (3)	C62—C63	1.533 (3)
C31—P1	1.840 (2)	C62—H62A	0.97
C31—H31	0.98	C62—H62B	0.97
C32—C33	1.539 (3)	C63—C64	1.517 (3)
C32—H32A	0.97	C63—H63A	0.97
C32—H32B	0.97	C63—H63B	0.97
C33—C34	1.513 (4)	C64—C65	1.517 (4)
C33—H33A	0.97	C64—H64A	0.97
C33—H33B	0.97	C64—H64B	0.97
C34—C35	1.527 (4)	C65—C66	1.533 (3)
C34—H34A	0.97	C65—H65A	0.97
C34—H34B	0.97	C65—H65B	0.97
C35—C36	1.534 (3)	C66—H66A	0.97
C35—H35A	0.97	C66—H66B	0.97
C35—H35B	0.97	P1—Ag	2.4046 (5)
C36—H36A	0.97	P2—Ag	2.4303 (6)
C36—H36B	0.97	N—O3	1.239 (3)
C41—C46	1.394 (3)	N—O1	1.247 (3)
C41—C42	1.398 (3)	N—O2	1.265 (3)
C41—P2	1.832 (2)		
			
C16—C11—C12	118.95 (18)	C42—C43—H43	119.9
C16—C11—P1	123.54 (15)	C43—C44—C45	120.0 (2)
C12—C11—P1	117.10 (16)	C43—C44—H44	120
C13—C12—C11	119.6 (2)	C45—C44—H44	120
C13—C12—H12	120.2	C46—C45—C44	119.8 (2)
C11—C12—H12	120.2	C46—C45—H45	120.1
C14—C13—C12	120.7 (2)	C44—C45—H45	120.1
C14—C13—H13	119.6	C41—C46—C45	120.3 (2)
C12—C13—H13	119.6	C41—C46—H46	119.8
C13—C14—C15	120.02 (19)	C45—C46—H46	119.8
C13—C14—H14	120	C52—C51—C56	111.09 (18)
C15—C14—H14	120	C52—C51—P2	109.34 (15)
C14—C15—C16	119.2 (2)	C56—C51—P2	111.68 (15)
C14—C15—H15	120.4	C52—C51—H51	108.2
C16—C15—H15	120.4	C56—C51—H51	108.2
C11—C16—C15	121.30 (19)	P2—C51—H51	108.2
C11—C16—H16	119.4	C51—C52—C53	111.0 (2)
C15—C16—H16	119.4	C51—C52—H52A	109.4
C26—C21—C22	112.6 (2)	C53—C52—H52A	109.4
C26—C21—P1	116.35 (16)	C51—C52—H52B	109.4
C22—C21—P1	109.75 (16)	C53—C52—H52B	109.4
C26—C21—H21	105.8	H52A—C52—H52B	108
C22—C21—H21	105.8	C54—C53—C52	110.1 (3)
P1—C21—H21	105.8	C54—C53—H53A	109.6
C23—C22—C21	113.2 (2)	C52—C53—H53A	109.6
C23—C22—H22A	108.9	C54—C53—H53B	109.6
C21—C22—H22A	108.9	C52—C53—H53B	109.6
C23—C22—H22B	108.9	H53A—C53—H53B	108.2
C21—C22—H22B	108.9	C55—C54—C53	109.8 (2)
H22A—C22—H22B	107.7	C55—C54—H54A	109.7
C22—C23—C24	112.8 (2)	C53—C54—H54A	109.7
C22—C23—H23A	109	C55—C54—H54B	109.7
C24—C23—H23A	109	C53—C54—H54B	109.7
C22—C23—H23B	109	H54A—C54—H54B	108.2
C24—C23—H23B	109	C54—C55—C56	111.4 (2)
H23A—C23—H23B	107.8	C54—C55—H55A	109.3
C25—C24—C23	111.4 (2)	C56—C55—H55A	109.3
C25—C24—H24A	109.3	C54—C55—H55B	109.3
C23—C24—H24A	109.3	C56—C55—H55B	109.3
C25—C24—H24B	109.3	H55A—C55—H55B	108
C23—C24—H24B	109.3	C51—C56—C55	111.02 (19)
H24A—C24—H24B	108	C51—C56—H56A	109.4
C24—C25—C26	112.5 (2)	C55—C56—H56A	109.4
C24—C25—H25A	109.1	C51—C56—H56B	109.4
C26—C25—H25A	109.1	C55—C56—H56B	109.4
C24—C25—H25B	109.1	H56A—C56—H56B	108
C26—C25—H25B	109.1	C62—C61—C66	110.74 (18)
H25A—C25—H25B	107.8	C62—C61—P2	116.34 (15)
C21—C26—C25	113.1 (2)	C66—C61—P2	108.02 (15)
C21—C26—H26A	109	C62—C61—H61	107.1
C25—C26—H26A	109	C66—C61—H61	107.1
C21—C26—H26B	109	P2—C61—H61	107.1
C25—C26—H26B	109	C61—C62—C63	111.82 (19)
H26A—C26—H26B	107.8	C61—C62—H62A	109.3
C36—C31—C32	110.69 (18)	C63—C62—H62A	109.3
C36—C31—P1	110.06 (15)	C61—C62—H62B	109.3
C32—C31—P1	111.08 (15)	C63—C62—H62B	109.3
C36—C31—H31	108.3	H62A—C62—H62B	107.9
C32—C31—H31	108.3	C64—C63—C62	111.7 (2)
P1—C31—H31	108.3	C64—C63—H63A	109.3
C31—C32—C33	110.64 (19)	C62—C63—H63A	109.3
C31—C32—H32A	109.5	C64—C63—H63B	109.3
C33—C32—H32A	109.5	C62—C63—H63B	109.3
C31—C32—H32B	109.5	H63A—C63—H63B	107.9
C33—C32—H32B	109.5	C65—C64—C63	111.3 (2)
H32A—C32—H32B	108.1	C65—C64—H64A	109.4
C34—C33—C32	111.1 (2)	C63—C64—H64A	109.4
C34—C33—H33A	109.4	C65—C64—H64B	109.4
C32—C33—H33A	109.4	C63—C64—H64B	109.4
C34—C33—H33B	109.4	H64A—C64—H64B	108
C32—C33—H33B	109.4	C64—C65—C66	111.1 (2)
H33A—C33—H33B	108	C64—C65—H65A	109.4
C33—C34—C35	111.6 (2)	C66—C65—H65A	109.4
C33—C34—H34A	109.3	C64—C65—H65B	109.4
C35—C34—H34A	109.3	C66—C65—H65B	109.4
C33—C34—H34B	109.3	H65A—C65—H65B	108
C35—C34—H34B	109.3	C65—C66—C61	111.5 (2)
H34A—C34—H34B	108	C65—C66—H66A	109.3
C34—C35—C36	110.5 (2)	C61—C66—H66A	109.3
C34—C35—H35A	109.6	C65—C66—H66B	109.3
C36—C35—H35A	109.6	C61—C66—H66B	109.3
C34—C35—H35B	109.6	H66A—C66—H66B	108
C36—C35—H35B	109.6	C11—P1—C31	104.92 (10)
H35A—C35—H35B	108.1	C11—P1—C21	103.60 (10)
C31—C36—C35	111.56 (19)	C31—P1—C21	106.42 (10)
C31—C36—H36A	109.3	C11—P1—Ag	110.98 (7)
C35—C36—H36A	109.3	C31—P1—Ag	114.74 (7)
C31—C36—H36B	109.3	C21—P1—Ag	115.12 (7)
C35—C36—H36B	109.3	C41—P2—C51	104.07 (9)
H36A—C36—H36B	108	C41—P2—C61	105.19 (9)
C46—C41—C42	119.01 (19)	C51—P2—C61	107.77 (10)
C46—C41—P2	123.32 (16)	C41—P2—Ag	113.46 (7)
C42—C41—P2	117.66 (16)	C51—P2—Ag	113.33 (7)
C43—C42—C41	120.6 (2)	C61—P2—Ag	112.34 (7)
C43—C42—H42	119.7	P1—Ag—P2	154.662 (19)
C41—C42—H42	119.7	O3—N—O1	120.5 (2)
C44—C43—C42	120.2 (2)	O3—N—O2	121.4 (2)
C44—C43—H43	119.9	O1—N—O2	118.1 (2)
			
C16—C11—C12—C13	3.5 (4)	C64—C65—C66—C61	55.6 (3)
P1—C11—C12—C13	176.4 (2)	C62—C61—C66—C65	−55.0 (3)
C11—C12—C13—C14	−4.3 (4)	P2—C61—C66—C65	176.6 (2)
C12—C13—C14—C15	4.9 (4)	C16—C11—P1—C31	−39.6 (2)
C13—C14—C15—C16	−4.7 (4)	C12—C11—P1—C31	147.89 (18)
C12—C11—C16—C15	−3.4 (4)	C16—C11—P1—C21	71.9 (2)
P1—C11—C16—C15	−175.79 (19)	C12—C11—P1—C21	−100.68 (19)
C14—C15—C16—C11	4.0 (4)	C16—C11—P1—Ag	−164.03 (18)
C26—C21—C22—C23	−49.1 (3)	C12—C11—P1—Ag	23.4 (2)
P1—C21—C22—C23	179.65 (17)	C36—C31—P1—C11	−66.86 (17)
C21—C22—C23—C24	51.1 (3)	C32—C31—P1—C11	170.20 (16)
C22—C23—C24—C25	−53.0 (3)	C36—C31—P1—C21	−176.26 (15)
C23—C24—C25—C26	53.3 (3)	C32—C31—P1—C21	60.80 (18)
C22—C21—C26—C25	49.5 (3)	C36—C31—P1—Ag	55.19 (15)
P1—C21—C26—C25	177.35 (16)	C32—C31—P1—Ag	−67.75 (17)
C24—C25—C26—C21	−52.2 (3)	C26—C21—P1—C11	−59.47 (17)
C36—C31—C32—C33	55.8 (3)	C22—C21—P1—C11	69.78 (18)
P1—C31—C32—C33	178.34 (18)	C26—C21—P1—C31	50.86 (18)
C31—C32—C33—C34	−56.2 (3)	C22—C21—P1—C31	−179.89 (16)
C32—C33—C34—C35	56.5 (3)	C26—C21—P1—Ag	179.19 (13)
C33—C34—C35—C36	−55.8 (3)	C22—C21—P1—Ag	−51.57 (17)
C32—C31—C36—C35	−56.0 (2)	C46—C41—P2—C51	−37.0 (2)
P1—C31—C36—C35	−179.19 (16)	C42—C41—P2—C51	143.49 (17)
C34—C35—C36—C31	55.6 (3)	C46—C41—P2—C61	76.23 (19)
C46—C41—C42—C43	−0.3 (3)	C42—C41—P2—C61	−103.30 (17)
P2—C41—C42—C43	179.29 (17)	C46—C41—P2—Ag	−160.60 (15)
C41—C42—C43—C44	1.1 (3)	C42—C41—P2—Ag	19.87 (18)
C42—C43—C44—C45	−0.5 (3)	C52—C51—P2—C41	−62.04 (19)
C43—C44—C45—C46	−1.0 (3)	C56—C51—P2—C41	174.61 (16)
C42—C41—C46—C45	−1.2 (3)	C52—C51—P2—C61	−173.38 (17)
P2—C41—C46—C45	179.25 (16)	C56—C51—P2—C61	63.27 (18)
C44—C45—C46—C41	1.9 (3)	C52—C51—P2—Ag	61.67 (18)
C56—C51—C52—C53	−55.5 (3)	C56—C51—P2—Ag	−61.68 (17)
P2—C51—C52—C53	−179.2 (2)	C62—C61—P2—C41	−65.12 (17)
C51—C52—C53—C54	58.0 (3)	C66—C61—P2—C41	60.08 (18)
C52—C53—C54—C55	−58.9 (3)	C62—C61—P2—C51	45.45 (18)
C53—C54—C55—C56	58.1 (3)	C66—C61—P2—C51	170.66 (16)
C52—C51—C56—C55	53.9 (3)	C62—C61—P2—Ag	171.00 (13)
P2—C51—C56—C55	176.25 (16)	C66—C61—P2—Ag	−63.80 (17)
C54—C55—C56—C51	−55.7 (3)	C11—P1—Ag—P2	100.90 (8)
C66—C61—C62—C63	54.3 (3)	C31—P1—Ag—P2	−17.80 (9)
P2—C61—C62—C63	178.13 (16)	C21—P1—Ag—P2	−141.85 (8)
C61—C62—C63—C64	−54.7 (3)	C41—P2—Ag—P1	29.07 (9)
C62—C63—C64—C65	54.9 (3)	C51—P2—Ag—P1	−89.33 (8)
C63—C64—C65—C66	−55.4 (3)	C61—P2—Ag—P1	148.22 (7)
